# The Effect of Temperature and UV Manipulation on Anthocyanins, Flavonols, and Hydroxycinnamoyl-Tartrates in cv Nebbiolo Grapes (*Vitis vinifera* L.)

**DOI:** 10.3390/plants13223158

**Published:** 2024-11-10

**Authors:** Alena Wilson, Alessandra Ferrandino, Simone Giacosa, Vittorino Novello, Silvia Guidoni

**Affiliations:** Department of Agricultural, Forest and Food Sciences, Università degli Studi di Torino, Largo Braccini 2, 10095 Grugliasco, Italy; alessandra.ferrandino@unito.it (A.F.); simone.giacosa@unito.it (S.G.); vittorino.novello@unito.it (V.N.)

**Keywords:** climate change, adaptation, fruit quality, polyphenols, terroir

## Abstract

This research aimed to identify the effects of increased temperature and decreased ultraviolet (UV) exposure on berry characteristics and quality parameters of cv Nebbiolo, identifying the potential risks associated with climate change for the quality of grapes and the identity of Barolo wine. This two-year research (2022 and 2023) was performed in three vineyards, located at different elevations in La Morra (Piedmont, northwestern Italy), monitored from the beginning of veraison to harvest. A split-plot design was set up, applying a passive greenhouse amplifying temperature in the bunch zone (‘T’ = increased temperature; ‘C’ = control temperature) and UV-blocking plastics over individual bunches (‘1’ = full UV exposure; ‘0’ = UV-blocked). Berry weight, skin weight, and juice total soluble solids were measured. Grape skin anthocyanins, flavonols, and hydroxycinnamic acid tartaric esters were analyzed by HPLC-DAD. Both treatments negatively influenced the berry weight but not the skin weight; the increased T had a negative impact on the sugar per berry content. Limited UV and increased temperature negatively impacted total anthocyanins at harvest and on di-hydroxylated anthocyanins. Limited UV-depressed flavonol concentration and high temperature decreased their synthesis. Increased UV promoted *cis-p*-coumaroyl tartaric acid and decreased *trans-p*-coumaroyl tartaric acid. The results of this research are valuable for improving the quality production of Nebbiolo through understanding the impacts of increased temperature and alterations to UV exposure expected by climate change.

## 1. Introduction

The concept of terroir is complex and can include impacts from the natural environment such as soil type, climate, geomorphology, and geology on grapes and wine [1,2]. With a changing climate, the risk of loss of association of a wine to its terroir is a concern. Many of the subtle differences in the color, flavor, and texture of a cultivar and ultimately a wine produced in different terroirs are associated with polyphenols, predominantly accumulated in the berry skin. This is especially true for red wines, which undergo skin contact during fermentation to extract polyphenols and improve wine quality. Although berry polyphenols have a genetic signature, their evolution and relative abundance can largely be altered by environmental factors [3,4,5,6].

It is well established that temperatures are increasing globally, as published by the Intergovernmental Panel on Climate Change (IPCC), with current (2011–2020) observed increases in average global temperatures of 1.1 °C above levels from the years between 1850–1900 [7]. Heatwaves have also been observed to increase in frequency, intensity, and duration [7,8]. Effects on ultraviolet (UV) radiation from climate change are less clear. The amount of UV radiation reaching the earth’s surface is influenced by changes in stratospheric ozone, with a decrease in ozone leading to increased UV-B. UV radiation reaching the earth’s surface can also be reduced by climate change from increased cloud cover, pollution, dust, smoke, and other particles [9]. The IPCC currently considers there to be a medium level of confidence that southern Europe will observe increased UV radiation, while the confidence in increased UV radiation in northern Europe is low [9].

UV-B radiation may modify the quantitative and qualitative profile of grape skin flavonols and may enhance extractable anthocyanins, as was observed in cv Tempranillo [10]. UV-B can promote the accumulation of phenolic acids, stilbenes, and flavonoids in grapevine leaves as an acclimation and protective response. High UV-B applications also increased total phenols in grape berries and, in particular, di-hydroxylated anthocyanins and flavonols like quercetin [11].

Anthocyanins predominantly accumulate in the grape skins of colored grapes. They are the primary source of color in red wines, and they accumulate following the expression of the gene coding for UDP-glucose:flavonoid 3-*O*-glucosyl transferase (UFGT) at veraison [4,12,13]. The anthocyanin profile and concentration in grape skins mainly depend on the variety [5,14] but also on berry temperature, solar radiation exposure [3,15,16,17,18,19], and water availability [20,21]. In many grapevine varieties, malvidin 3-*O*-glucoside is the predominant anthocyanin [5,14]. Nebbiolo-based wines are known for having weak color, which can be associated with a generally low content of anthocyanins in the berry skins, and a prevalence of di-hydroxylated forms [3,22]. Di-hydroxylated anthocyanins are not as stable in wine as tri-hydroxylated anthocyanins, which is a second reason for weak color in Nebbiolo-based wines.

Flavonols act as a primary defense against UV exposure in vegetal tissues [5,13,16,23,24]. They accumulate in berry skin, and their synthesis is stimulated by exposure to solar radiation and UV [25,26]. For this reason, agronomical practices that increase bunch exposure to solar radiation, as well as a natural low vigor of the vines, increase polyphenol concentration in the skin, particularly that of flavonols [27,28]. On the contrary, bunch shading has a detrimental effect on the synthesis of flavonols in the berry [17,29,30,31,32]. Flavonol accumulation peaks twice during berry development. The first peak occurs at flowering, and the second occurs approximately 3 to 4 weeks after veraison [5]. The impact of increased temperature on flavonol concentrations is not clear, with some research suggesting that temperature has little to no effect [33], whereas others observed a significant decrease in flavonol concentration with the application of very high temperatures (>50 °C) for 12 h [34]. Flavonols are perceived as a quality enhancer in wine partly due to their ability to stabilize anthocyanin color through co-pigmentation [35,36]. Further, some flavonols are associated with a bitter flavor, which is believed to enhance quality perception [37]. Flavonols are often found in higher concentrations in premium wines due to the practice of leaf removal, which is used to increase airflow and dry the grapes, protect them against disease, and increase exposure for treatment application in the bunch zone. Beyond the concern of loss of terroir identity, there are also new risks associated with some of these polyphenols. Specifically, the flavonol quercetin has been increasing in concentration in some regions, including Tuscany [38]. Higher concentrations of quercetin can lead to deposits and increased turbidity in bottled wines, resulting in negative quality perception [39]. Further, recent research suggests that quercetin 3-*O*-glucuronide could be the main culprit associated with headaches from red wine consumption [40].

Hydroxycinnamic acids esterified with tartaric acid (HCTA) are non-flavonoid compounds found in grape berries and wines. HCTAs display the highest concentration of non-flavonoid compounds in berries and accumulate in berry skin and pulp [41]. HCTAs are reported as UV-B-absorbing phenols in the leaves [42], where they were found to be unaffected or positively influenced by visible radiation [43]. Still, there is limited information about their accumulation in berries and the environmental factors that can influence their concentrations and profiles. In white varieties, hydroxycinnamic acids and their derivatives were negatively influenced by UV exposure [26]. In the colored skins of Cabernet Sauvignon grapes, UV deprivation slightly reduced the concentration of HCTA [23]. The increase in cluster exposure to light, associated with leaf removal, significantly increased the concentration of HCTA in the berry skins of Tempranillo [44] and of Istrian Malvasia [45].

In red wines, HCTAs are known to stabilize color and can also impart a bitter flavor; thus, they have been associated with increased quality perception [46,47]. Additionally, they have garnered attention for their potential health benefits, acting as antioxidants with possible implications in reducing the risk of Alzheimer’s, Parkinson’s, cardiovascular disease, and diabetes [48]. HCTAs also pose some risks to wine quality that may offset their potential benefits. Specifically, *p*-coumaric and ferulic acid esters can be metabolized by *Brettanomyces* and *Dekkera bruxellensis* yeast species to ethylphenols, which can significantly lower the quality of a wine’s aroma and flavor [49]. In previous research, it was shown that reducing the concentration of HCTAs and of the relative cinnamic acid could significantly reduce the concentration of 4-vinylguaiacol and 4-vinylphenol, notably responsible for the ‘brett’ aroma of wines [50].

The aim of this two-year research was to identify the effects of artificially altered exposure to UV radiation and temperature on total and individual anthocyanins, flavonols, and hydroxycinnamates in the skins of cv Nebbiolo grapes (*Vitis vinifera* L.) under field conditions. The artificial temperature amplification and UV limitation were intended to emulate severe conditions associated with climate change in order to determine potential risks to berry quality and associated terroir.

## 2. Results and Discussion

### 2.1. Efficacy of Treatment Factors

#### 2.1.1. Greenhouse Plastic

**Figure 1:**
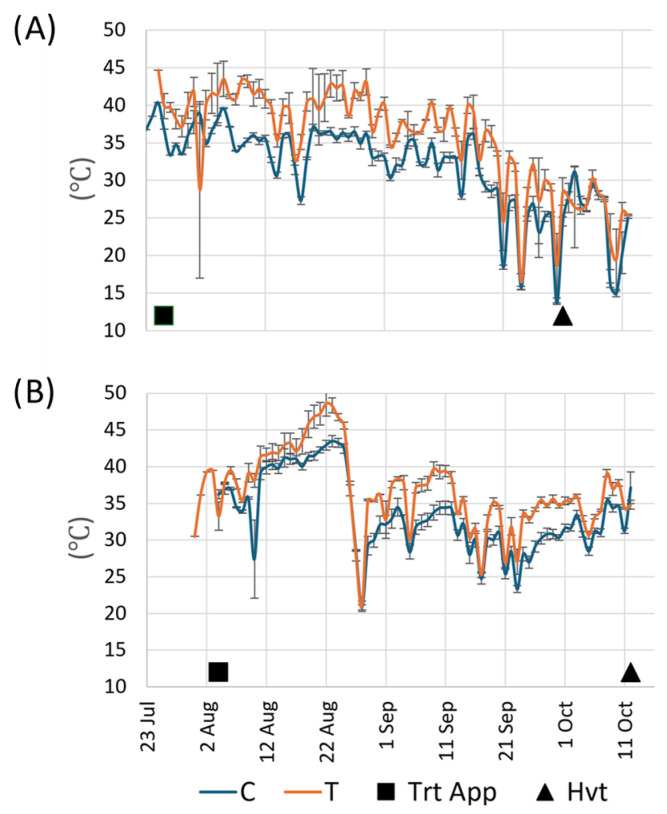
Average maximum daily temperature in vineyards of C (blue) and T treatments (orange) in 2022 (**A**) and in 2023 (**B**). Data expressed as mean values ± standard errors. Black square (■) indicates treatment application (TrtApp) date. Black triangle (▲) indicates harvest (Hvt) date.

As expected, the passive greenhouse affected a daily increase in temperature for T-treated vines compared to C-treated vines during the season, between 2 and 7 °C, with a duration of 4 to 6 h during days with full sun. The daily average maximum temperature in T vines of all vineyards in the month of September was 5.1 °C higher than in C vines in 2022 and 4.6 °C higher than C in 2023 (Figure 1).

#### 2.1.2. UV-Block

The measurements of penetrative photosynthetically active radiation (PAR), UVA, and UVB confirm the efficacy of the UV-blocking plastic in reducing UV radiation, especially UVB. It also emerged that greenhouse plastic partially reduced the UVA, UVB, and PAR by 24%, 32%, and 14%, respectively (Table 1).

To confirm that UV-blocking plastic treatment was not increasing temperatures, a one-way ANOVA was performed on hourly temperature measurements in C1 and C0 treatments between May and July of 2022. Results showed no significant difference.

### 2.2. Precipitation, Soil Volumetric Water Content (SWC) and Soil Temperature (SoilT)

**Figure 2:**
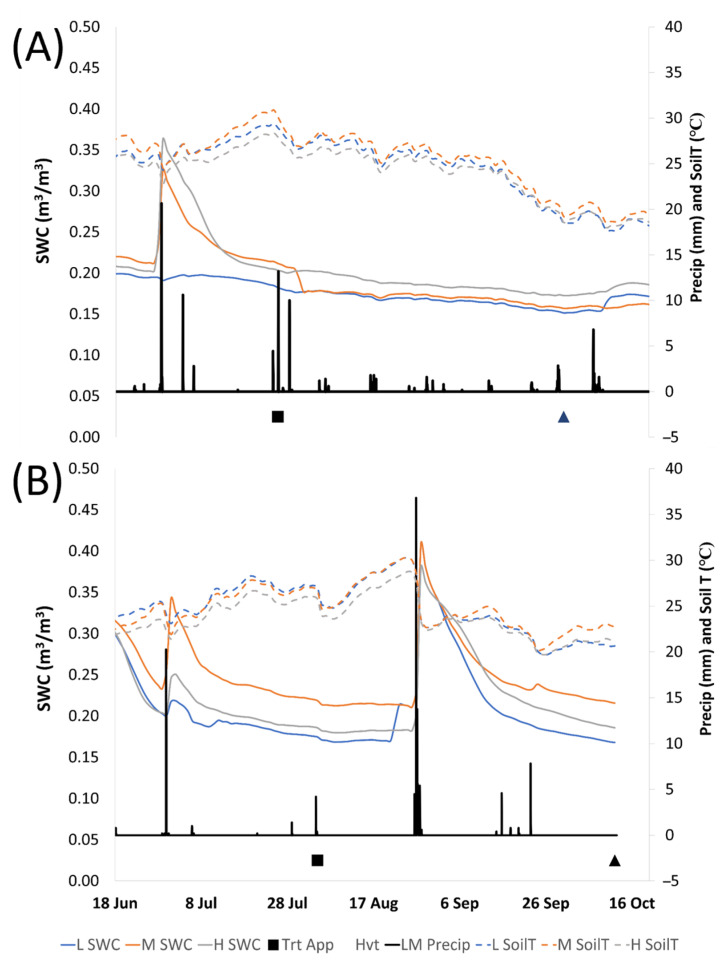
Mean daily average Soil Water Content (SWC, continuous lines) and Soil Temperature (SoilT, dashed lines) at 30 cm depth in the three studied vineyards. Precipitation (Precip) was measured from the La Morra weather station (black line) during the berry ripening period in 2022 (**A**) and 2023 (**B**). The black square indicates the date of treatment application (Trt App). The black triangle indicates the average harvest date (Hvt).

The 2022 growing season was characterized by low precipitation. At the La Morra weather station, precipitation reached 432 mm. In contrast, 2023 experienced significantly higher precipitation, with an annual total amount of 631 mm. The most notable disparity occurred from April to June, indicating a wetter spring in 2023 compared to 2022. From the treatment application until harvest, 27 rainfall events resulted in 88 mm in 2022, with one event of 34 mm occurring between July 26 and July 29, and no events above 10 mm observed after this moment until harvest. In 2023, from treatment application to harvest, 11 events caused 150 mm of rain, with a significant rainfall (97 mm in 11 h) occurring on August 27–28 (Figure 2).

At the beginning of the observation in 2022, the SWC was between 0.20 and 0.22 m^3^/m^3^ in all vineyards. During the 2022 season, SWC increased to 0.30 and 0.35 m^3^/m^3^ in M and H, respectively, after the rainfall on June 28 (38 mm in two hours). After this, it declined slowly and stabilized (Figure 2A). There was no observed increase in SWC in vineyard L associated with this rainfall. A series of rainfall events occurred from July 27 to 29 at the approximate time of treatment application; however, these events did not increase SWC in any vineyard (Figure 2A). All vineyards remained between 0.16 and 0.20 m^3^/m^3^ SWC for the rest of the season. In the spring of 2023, several rainfall events led to a SWC of 0.3 m^3^/m^3^ on June 18, which declined until the rainfall on June 29 (Figure 2B). The SWC reached slightly lower values than those after the June rainfall of 2022, with a peak of 0.34 m^3^/m^3^ at vineyard M, while in both H and L, the SWC reached lower values of 0.25 m^3^/m^3^ and 0.22 m^3^/m^3^, respectively. The second rainfall event occurred on August 27–28 and caused an increase in SWC to between 0.38 and 0.41 m^3^/m^3^, depending on the vineyard. SWC appeared to increase significantly after each summer precipitation event greater than 15 mm. During the 2023 season, SWC remained above 0.19 m^3^/m^3^ in H and M while dropping to 0.17 m^3^/m^3^ in vineyard L at harvest (Figure 2B). In both years, vineyard L had the lowest SWC, while H had the highest in 2022 and M was the highest in 2023. However, at the end of the observation period, the mean SWC was comparable between years, especially for L and H vineyards. Low volumes of more frequently distributed rainfall, as observed in 2022, appear to maintain an SWC equal to periods receiving fewer events of higher volumes (as seen in 2023).

Soil temperature at 30 cm depth showed a peak on 25 July 2022, immediately prior to the main rainfall event. This rainfall did not reflect a change in SWC but did appear to reduce SoilT (Figure 2A). Afterward, SoilT stabilized between 24.0 °C and 26.0 °C until September 18, after which a continuous decline occurred until it reached 19.0 °C at harvest. The SoilT trend differed in 2023, with the maximum value of 29.7 °C occurring between August 24 and 27, prior to the major rainfall event, which led to a notable decline to 23.6 °C on August 28. The SoilT stabilized between 21.0 and 23.0 °C in H and L vineyards until harvest, whereas a slight increase was observed in vineyard M until harvest. In both years, vineyards H and L trended lower in SoilT as compared to vineyard M. Observations have shown that more evenly distributed rainfall, even when less abundant (as in 2022), was more effective at maintaining soil temperature at a constant or lower level with respect to higher amounts of precipitation concentrated in specific moments (as in 2023).

### 2.3. Air Temperature

In 2022, the HI of C treatment in vineyard M (at 360 m ASL) was higher than L (210 m ASL) and H (410 m ASL) (Table 2). However, in 2023, a reversal was observed, with L and H having a higher HI than M. The HI was generally higher in 2023 than in 2022, but in both seasons, the differences between the HI of treatment T and that of treatment C were similar for all vineyards, with T higher than C, as expected (Table 2). However, the HI does not capture the whole picture as it does not consider short-term extreme temperature conditions. For this reason, the number of hours with temperatures above 35, 40, and 45 °C were also calculated between August 4 and the harvest date in both years. T treatment amplified the number of hours of exposure to high temperatures in all vineyards in both years. The generally higher temperatures (and HI) of 2023 were reflected in the greater number of hours with temperatures above 35 °C in C treatment and above 40 and 45 °C in T treatment. Notably, no vineyard had any hours with temperatures above 45 °C in either year in the C treatment, while in the T treatment, L vineyard reached 11 h in 2022 and 37 h in 2023, M vineyard experienced 0 h in 2022 and 4 h in 2023, and H vineyard had 2 h above 45 °C in 2022 and 12 h in 2023 (Table 2).

### 2.4. Berry Characteristics

**Figure 3:**
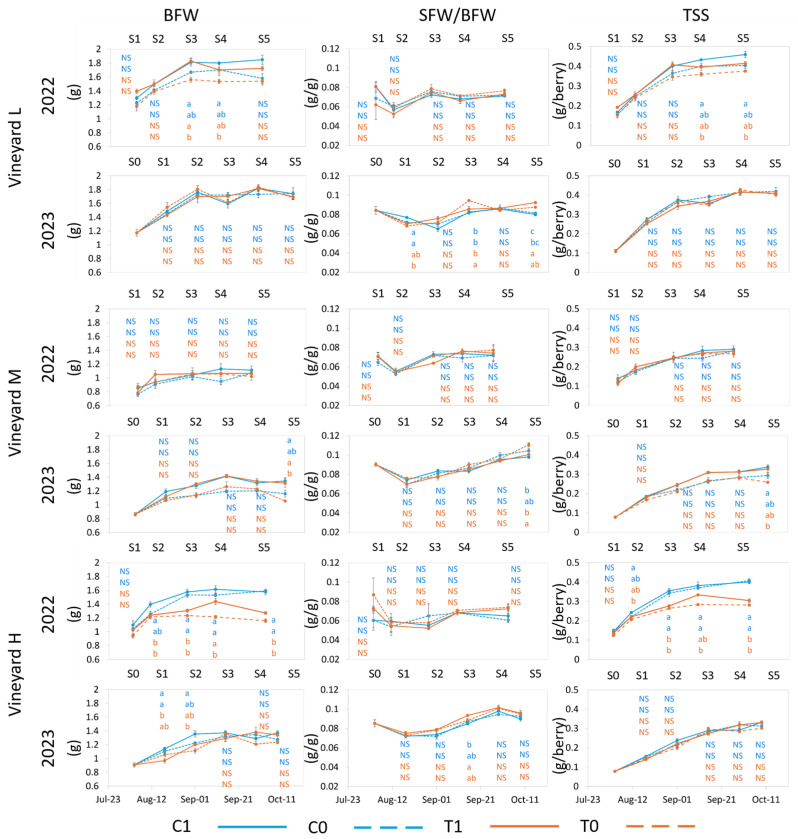
Evolution of berry fresh weight (BFW), fresh skin weight/fresh berry weight ratio (SFW/BFW), and total soluble solids (TSS, g/berry) for each vineyard from treatment application to harvest in 2022 and 2023. Error bars represent standard errors (*n* = 3 and *n* = 4 for C1 in 2023). Different letters indicate significant differences for *p* ≤ 0.05. The letters are presented vertically in order: C1, C0 (blue color), T1, T0 (orange color). S0–S5 represent sample points.

Berry weight at harvest was influenced by season and vineyards, being the highest in vineyard L in both years and the lowest in vineyard M in 2022 and in vineyard H in 2023, respectively (Figure 3). In vineyard L, no difference in berry weight at harvest was evident among treatments in either year, although in 2022, at S3, berries exposed to UV (C1 and T1) reached higher berry weights than those of treatment T0, and at S4, C1 had a higher berry weight than T0. At harvest (S5), although C1 and T1 trended higher than C0 and T0, there was no significant difference. Berry weight between treatments in vineyard M was not different at any sample point in 2022; at harvest in 2023, berries from C1 and T1 treatments displayed significantly higher weight than T0, while C0 was intermediate. In berries from vineyard H, a clear and significant separation among grapes exposed to ambient temperature (C1 and C0) compared to those under increased temperature (T1 and T0) started at S2 in 2022, while in 2023, although there were differences at S2 and S3 between C and T-treated berries, no significant difference was maintained at harvest (Figure 3). Higher thermal accumulations (vineyard L and H in 2023 and vineyard M in 2022) caused the berries to have a smaller mass, but with very few differences between treatments. Berry weight displayed greater differences among treatments in the less stressful thermal conditions, seen in 2022 for vineyards L and H and in 2023 for vineyard M (Table 2, Figure 3). This suggests that berry weight can vary more greatly when berries are exposed to increased temperature if ambient conditions are cooler rather than in years with higher ambient temperature. Therefore, the potential exists to use vineyard management techniques to increase temperature and decrease berry weight in cooler years, but these techniques may not be as effective in warmer seasons. On the other hand, in years with extreme temperatures, efforts to reduce heat stress may not have a significant impact on berry size or yield.

Despite the small and inconsistent differences among treatments and between years, it seems that the temperature increase associated with T treatment had a greater negative impact on the berry weight than the UV block (treatment C0). This differs from other studies reporting no changes in berry weight with increased temperature [51] or reduced sun exposure [17] or reporting an increase in berry volume and weight when solar UV-B was filtered from flowering to harvest in cv Malbec [52]. These current results agree with those found in a study on cv Nebbiolo that reported a lower berry mass in warmer seasons or vineyards [53]. The treatments slightly influenced the skin weight, but season and vineyard had a higher impact on skin weight, with vineyard L having a higher skin weight than vineyards M and H (Supplementary Table S1). The skin-to-berry ratio was significantly higher in 2023 than in 2022 at harvest; however, it showed only slight differences among treatments during both seasons (Figure 3). No differences were found at any sample point or vineyard in 2022. In 2023, in vineyard L, berries that were grown under increased temperature (T1 and T0) had a higher skin-to-berry ratio when compared to C1 and C0. Since the berry weight was similar among treatments, this was due to the increase in skin weight as a response to the increased temperature or, as found on cv Malbec, to the decrease in UV radiation intensity [52]. In vineyard M, berries with full exposure to UV and ambient temperature (C1), as well as full UV exposure and amplified temperature (T1), displayed a lower ratio compared to T0, with C0 being intermediate. This response was more clearly associated with an increase in berry weight when exposed to UV. In vineyard H, no significant difference was found at harvest in both years. However, in 2023, T1 trended higher than C1 throughout the season, with significant differences at S3. The differences between years and vineyards and their interaction were significant for skin-to-berry ratio and skin weight (Supplementary Table S1). Generally, this ratio was altered by a reduction in berry weight observed with exposure to increased temperature, while skin weights were not altered meaningfully by treatments (Supplementary Table S1). Increased skin-to-berry weight is a quality parameter leading to increased phenolics in wine. However, the potential negative impacts that can arise from berry exposure to extreme temperatures may outweigh any potential quality enhancement from amplifying this ratio.

TSS per berry showed variation among vineyards and between seasons but limited variations among treatments. TSS (grams per berry) reflected the berry mass, and the highest content at harvest was found in the heaviest berries (vineyard L in both years) and the lowest in the smaller berries, in vineyard M in 2022 and in vineyard H in 2023. In the L vineyard, higher sugar content per berry was observed in C1 berries as compared to T0 berries in 2022 at S4 and S5. In 2023, no significant difference was observed at any sample point in vineyard L or H. In vineyard M, significant differences between grapes grown with full UV exposure (C1) and those with no UV exposure and increased temperature (T0) emerged at harvest in 2023 (Figure 3). In 2022, in vineyard H, berries grown under ambient temperature (C0 and C1) had a significantly higher per berry sugar content with respect to those grown under increased temperature (T1 and T0). This difference was significant from S2 to S5. The TSS was similar for all treatments when HI was highest (vineyards L and H in 2023 and vineyard M in 2022, Table 2), but it was, on average, lower than that achieved in the less warm conditions (Figure 3). These observations support previous findings wherein TSS accumulation was inhibited by exposure to high temperature (>40 °C) [53]. T treatments have, therefore, shown the same negative impact as ambient temperatures in a warmer year, suggesting that at a certain point, no further increased temperature or alteration of UV exposure will further reduce TSS. In all cases, extreme temperatures had a negative effect on the absolute value of TSS per berry. When significant differences were found among treatments, TSS was highest in C0 and lowest in T0, both as g/berry (Figure 3) and °Brix (Supplementary Table S1). A similar negative impact of filtered solar UV-B on soluble solids per berry was found in Malbec [52]. In all, it appears that berry characteristics are less elastic in conditions where they are exposed to high (>40 °C) temperatures, as was observed in vineyards H and L in 2023 and vineyard M in 2022. This suggests that in very hot years, there are fewer options available to producers to manipulate temperature and UV exposure to protect berry weight, skin-to-berry ratio, and TSS. However, in cooler years, berry characteristics may evolve differently depending on exposure to increased temperature and UV, which can be associated with canopy management techniques, row orientation, and slope aspect [1,3,53].

### 2.5. Anthocyanins

When expressed as mg/kg of fresh berries in vineyard L, the total anthocyanin concentration (TAC) at harvest was significantly influenced by the treatments in both years, with C0 berries reaching higher concentrations than both T treatments in 2022 and C1 being significantly higher than T0 in 2023. In vineyard M, although no significant differences were observed among treatments in either year at harvest, C1 berries reached the highest TAC in both years. In 2023, C0 and C1 both trended higher than T1 and T0. In vineyard H, C1 berries showed significantly higher concentrations than both T treatments in both years, with C0 also being significantly lower than C1 in 2023. The negative impact of high temperature on anthocyanin concentration has been shown in many studies in phytotron [19,54,55,56] and field conditions [17,57]. Azuma et al. (2012) [54] found reduced anthocyanin concentration and alterations to the profile under 35 °C due to changes in expression of flavonoid biosynthetic pathway genes.

**Figure 4:**
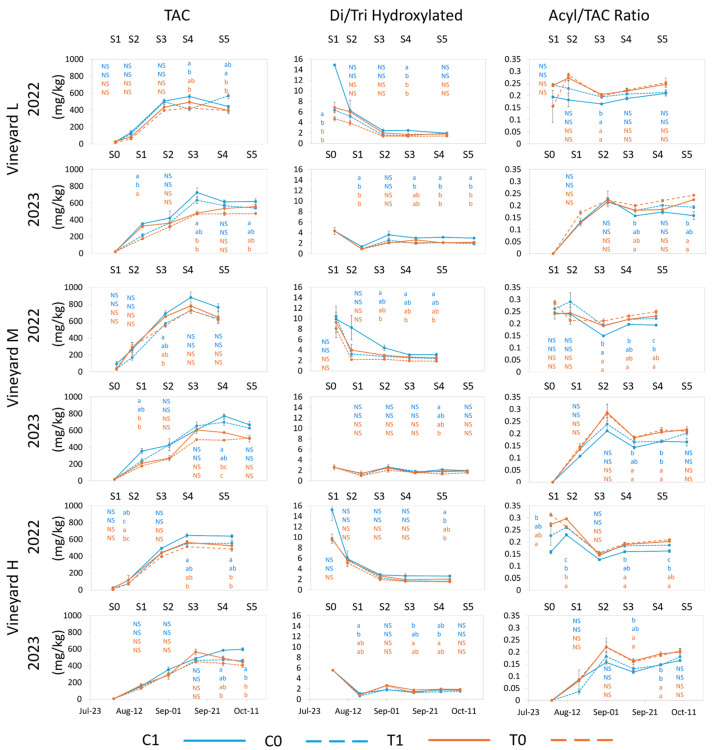
Evolution of total anthocyanin concentration (mg/kg) (TAC), di-hydroxylated/tri-hydroxylated anthocyanin ratio [Di/Tri = (Cn + Pn)/(Df + Pt + Mv)] and acylated/TAC ratio for each vineyard from treatment application to harvest in 2022 and 2023. Error bars represent standard error (*n* = 3 and *n* = 4 for C1 in 2023). Different letters within the same column indicate significant differences between treatments. *p* ≤ 0.05; NS = not significant. The letters are presented vertically in order: C1, C0 (blue color), T1, T0 (orange color). S0–S5 represent sample points.

UV treatments also appeared to reduce TAC in all vineyards with respect to C1 except for L vineyard in 2022 (Figure 4). This reduced concentration of TAC through the removal of UV appeared to be largely due to a response from the di-hydroxylated anthocyanins, which constitute the larger proportion of TAC in cv Nebbiolo. The concentration (Supplementary Table S2) and relative proportion (Supplementary Table S3) of di-hydroxylated anthocyanins were both negatively impacted by increased temperature but also by the removal of UV, which further amplified this effect (Supplementary Table S3). The relative proportion of the tri-hydroxylated anthocyanins (Mv in particular) was positively influenced by UV removal, whereas increased temperature had no effect on it (Supplementary Table S3). It has also been shown that the synthesis of anthocyanins can be depressed in the absence of light [54] but that high levels of radiation are not necessary for their synthesis, provided the temperature is not excessive [58].

The downregulation of F3′-5′H, associated with an increase in temperature, in a previous study resulted in a decrease in the percentage of tri-hydroxylated anthocyanins [54]. In the current study, this did not occur, and when UV exposure was reduced (C0), the percentage of Mv and Pt (Supplementary Table S3) increased even when temperature was artificially increased (T0). The relative abundance and concentration of Cy and Pn decreased due to high temperatures (T1) and, to a greater extent, in the absence of UV radiation (C0 and T0) (Supplementary Table S3). High temperatures and lack of UV appear to have had different effects on the di-hydroxylated and tri-hydroxylated anthocyanins. However, the ratio of di-hydroxylated to tri-hydroxylated anthocyanins decreased (as compared to C1) as a result of treatments. This was largely driven by alterations to the concentration of di-hydroxylated anthocyanins, with some differences at harvest among vineyards and year but not by treatment and year (Supplementary Table S4).

The rate of acylation is known to increase with increased temperature [16,54,56]. Similar findings were observed in this research consistently in each vineyard when comparing rates of acylation to TAC, with T treatments trending higher than C treatments in both years (Figure 4). The effect of UV on acylation rates was less clear, with two cases (vineyards M and H in 2022) showing higher values in C0 than in C1. However, in both of these cases, C0 was significantly lower than T0, suggesting that UV exposure plays less of a role in acylation rates than amplified temperature (Supplementary Table S4). In vineyard L, both T treatments had a higher ratio than C treatments (significantly higher in 2023) in both years. Vineyard M displayed significantly higher acylation rates in T0 than in C1 or C0 in 2022, while T1 was intermediate to C0 and T0. In 2023, C1 was lower than all other treatments, although, at harvest, differences were not significant. Finally, vineyard H also had significantly lower values for C1 treatment in 2022 compared to all other treatments, while C1 and C0 trended lower than T treatments in 2023 (Figure 4 and Supplementary Table S4). In conditions characterized by the high thermal accumulation in Nebbiolo berries, the synthesis of di-hydroxylated anthocyanins may decrease, especially if bunches are shaded from UV, and their acylation may increase, favoring the production of musts with a lower di/tri ratio and higher acylation rates. Considering that tri-hydroxylated and acylated anthocyanins are more stable than di-hydroxylated anthocyanins and their respective free forms, the increased temperature may alter the skin anthocyanin profile at harvest, potentially increasing, in a cv similar to Nebbiolo, the wine color stability over time, as previously reported [22]. In contrast to these findings, negative impacts of limited UV radiation on anthocyanin acylation have been reported [52,59], but the role of UV in this aspect would require more detailed investigations.

**Figure 5:**
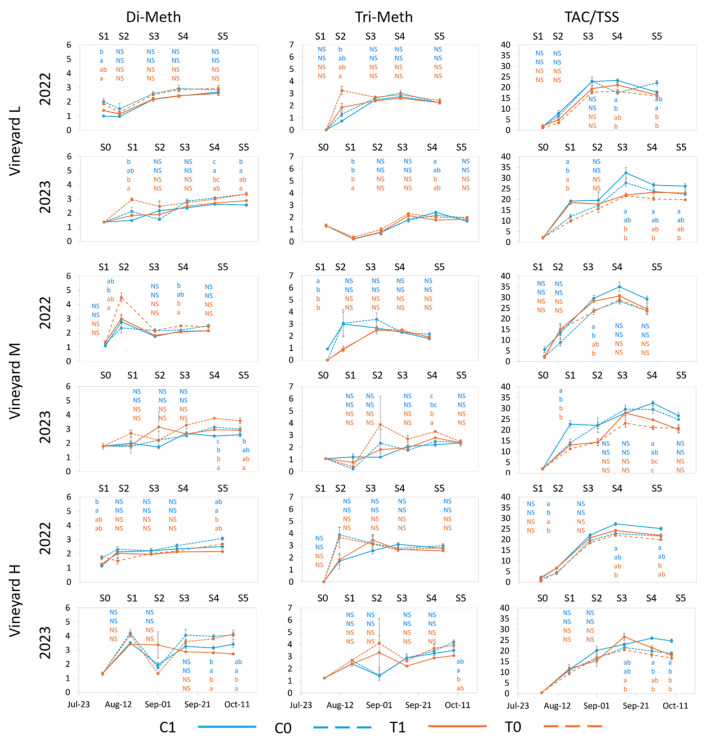
Evolution of Di-methylated ratio [Di-Meth = Pn/Cy], Tri-methylated ratio [Tri-Meth = Mv/(Df + Pt)], and TAC/TSS for each vineyard from treatment application to harvest in 2022 and 2023. Error bars represent standard error (*n* = 3 and *n* = 4 for C1 in 2023). Different letters within the same column indicate significant differences between treatments. *p* ≤ 0.05; NS = not significant. The letters are presented vertically in order: C1, C0 (blue color), T1, T0 (orange color). S0–S5 represent sample points.

Methylation of di-hydroxylated anthocyanins trended higher with limited UV exposure in all vineyards in both seasons (Figure 5, Supplementary Table S4). Significant differences were observed in all vineyards in 2023, with C0 and T0 generally having higher rates of methylation than C1 or T1, while in 2022, only vineyard H observed significant differences, with both UV-deprived treatments having higher rates of methylation of Pn and Cy than berries with UV exposure.

Throughout both seasons and at harvest, both treatment factors (UV and T) did not significantly influence the methylation of tri-hydroxylated anthocyanins, with the only exception being vineyard H in 2023, where C0 was significantly higher than T1. For both ratios, the relationship between treatments was inconsistent and did not exhibit a general trend (Figure 5), while significant differences were observed between years and vineyards (Supplementary Table S4). At 35 °C, a decrease in the methoxylated forms (Pn in particular) was previously observed due to the downregulation of O-methyltransferase [54], and an increase in the proportion of methoxylated forms was observed in cv Merlot with higher thermal regimes (day/night temperature = 30–35/20–30 °C) [56]. The current findings in cv Nebbiolo did not support these results.

The ratio TSS/TAC in 2022 and 2023 showed some significant difference between C and T treatments at harvest in both years, with C1 trending higher than both T treatments in both years and higher than C0 in vineyard H in 2023 (Figure 5). Until the beginning of September 2022, a linear increasing trend was observed, which was similar for all treatments and vineyards. After this point, TAC accumulated more slowly than TSS (ratio decreased), and accumulation was impacted by treatments with different trends depending on the vineyard and treatment. The decrease in the ratio prior to harvest in 2022 for T berries occurred despite the fact that during the two weeks prior to harvest, maximum temperatures were never above 35 °C in T treatments (Figure 1). This confirms the effect of high temperature on decoupling anthocyanin synthesis and/or accumulation and sugar accumulation as previously reported [15,60] and suggests that this can occur at temperatures lower than 35 °C or that impacts of higher temperature exposure, which occur earlier in the ripening period, can have prolonged consequences. Despite the lower temperatures observed during the period prior to harvest in 2022 as compared to temperatures prior to harvest in 2023, the SWC in 2022 was also much lower than in 2023 (Figure 1 and Figure 2). This difference in SWC between years could have influenced the impact of the treatments on this ratio and its seasonal trend. The pedoclimatic conditions were observed to alter TAC more than TSS (Figure 3 and Figure 4); however, in a hot year, such as 2023, increased SWC did not offset the effects of the high temperature on the decoupling of anthocyanins from sugar accumulation. Water deficit has previously been observed to increase the rate of anthocyanin accumulation and the ratio between TAC and TSS [60,61].

### 2.6. Flavonols

**Figure 6:**
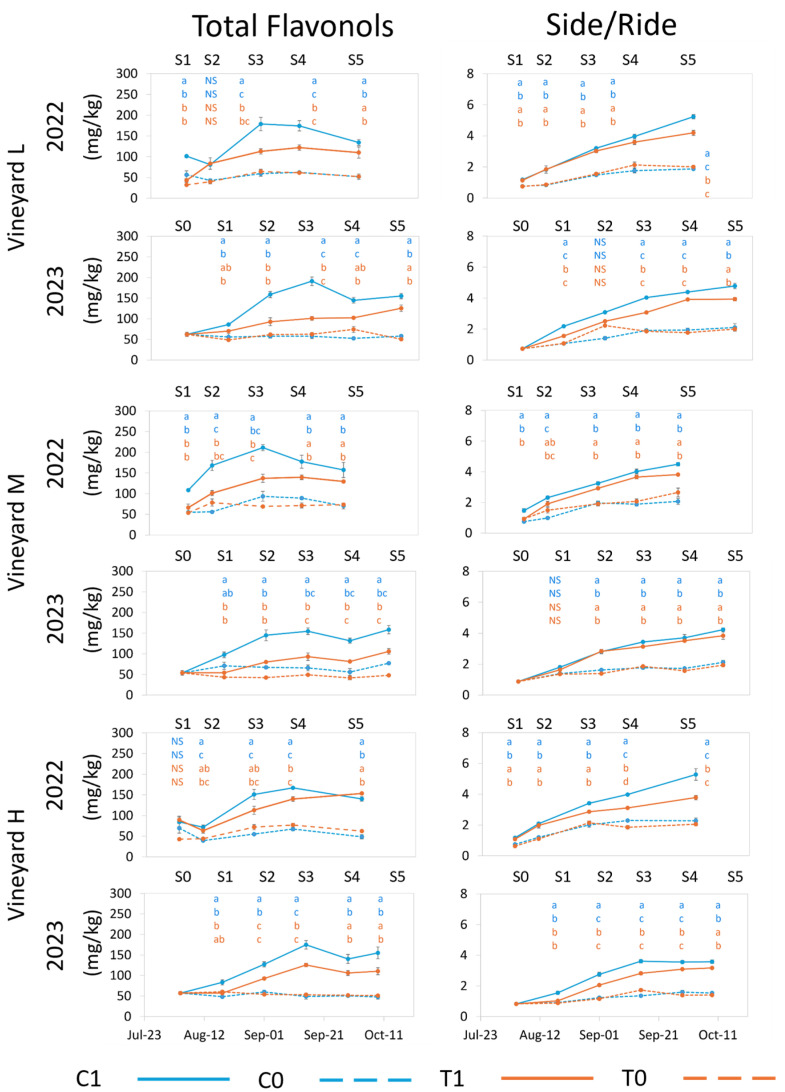
Evolution of total flavonols and glucoside/glucuronide (Side/Ride) ratio for each vineyard from treatment application to harvest in 2022 and 2023. Error bars represent standard error (*n* = 3 and *n* = 4 for C1 in 2023). Different letters within the same column indicate significant differences between treatments. *p* ≤ 0.05; NS = not significant. The letters are presented vertically in order: C1, C0 (blue color), T1, T0 (orange color). S0–S5 represent sample points.

Di-hydroxylated quercetin (Q) is the most abundant flavonol in Nebbiolo berries [14], where it is present in glucoside (Side) and glucuronide (Ride) forms. On average, in C1 and T1 samples, the glucoside form (QSide) was about four times more abundant than the glucuronide form (QRide) (Supplementary Tables S5 and S6). When UV radiation was limited (C0 and T0), the total flavonol concentration and individual molecule concentrations significantly decreased (Figure 6) in all vineyards and in both years, as expected. The QSide concentration decreased more than that of QRide (approximately 65% and 15%, respectively). However, kaempferol (K) glucoside and glucuronide decreased much more (about 85%) than Q forms (Supplementary Tables S5 and S6). This contributed to the increase in the proportion of QRide and MRide (Supplementary Table S5) and to the decrease in the ratio between glucoside and glucuronide forms (Side/Ride) at harvest in vines with reduced UV exposure (Supplementary Table S6). The Side/Ride ratio was highest in C1 and T1 and lowest in C0 and T0 from the start of the sampling after treatment application until harvest (Figure 6).

A positive response of flavonols to increased exposure to solar radiation has been observed multiple times in previous research [17,31,62,63,64]. The exclusion of UV has previously been observed to reduce the concentration of individual flavonols [10,11,54] while also modifying the relative abundance of the individual molecules. In Tempranillo, high doses of UV increased the relative abundance of the mono- and di-hydroxylated flavonols and decreased the proportion of tri-hydroxylated [10]. In our study, the concentration of total and individual flavonols decreased after reducing UV exposure (Supplementary Table S6), which was consistent with previous research. However, the proportion of myricetin glucoside (MSide) and quercetin glucuronide increased (Supplementary Table S5), confirming that limiting UV radiation can alter the flavonol profile.

The total concentration of flavonols and individual molecules decreased with increasing temperature. This decrease did not affect MSide and QRide under natural UV exposure (T1) (Supplementary Table S6), and therefore, their relative abundance increased when compared to C1 (Supplementary Table S5). This suggests that the molecules have a different heat sensitivity. The increased temperature had a much smaller effect on flavonol concentration compared to limited UV exposure and was, therefore, not compounding, meaning that the concentrations and relative abundance of the single molecules under T0 treatments remained similar to those of C0 but always lower than those of the control (C1). This agrees with a previous study that demonstrated that temperature negatively impacted total flavonol concentration in cv Merlot, particularly when the temperature was higher than 30 °C during the day and higher than 25 °C during the night [56]. A negative impact of temperatures higher than 35–40 °C has been observed in other studies [54,65,66]. One study observed a decrease in flavonol concentration only under extreme temperature environments (>50 °C) [16]. In T-treated vines, total flavonol concentration trended lower during both seasons, although at harvest, only in vineyard M in 2023 was the difference between C1 and T1 significant. Despite this, in 2023, the separation between C1 and T1 was more defined in all vineyards than in 2022 (Figure 6). This could be explained by the much higher number of hours T-treated vines were exposed to temperatures above 35 °C, 40 °C, and 45 °C in 2023 compared to 2022 (Table 2). This may suggest a non-linear relationship between temperature and flavonol synthesis or that this relation may be more influenced by prolonged temperatures above a certain threshold (35 °C in our case). Throughout the season, C1 berries contained significantly higher amounts of flavonols than T1 berries. However, at harvest, although C1 trended higher in all vineyards in both years, a significant difference between C1 and T1 occurred only in vineyard M in 2023 (Figure 6). However, under both ambient and increased temperatures, flavonol synthesis appeared to be completely depressed by the absence of UV. This is consistent with Azuma et al. (2012) [54], who found the influence of light on the expression of flavonol biosynthesis-related genes to be much more considerable than that of temperature.

### 2.7. HCTAs

**Figure 7:**
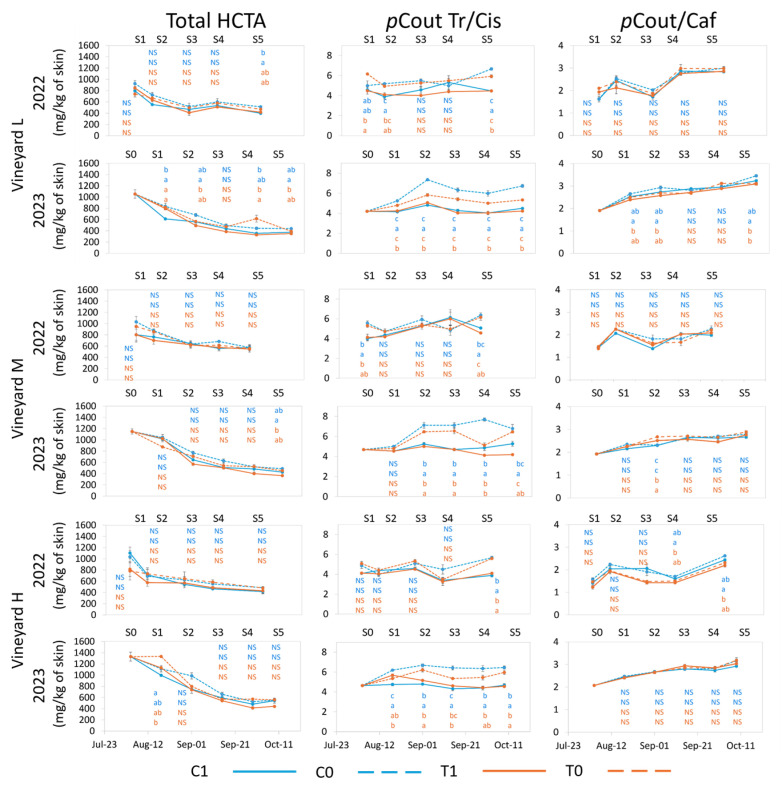
Evolution of Total HCTAs (mg/kg berry skins), ratio of *trans/cis p*-Coumarylated (*p*Cout Tr/Cis), and *p*-Coum/Caftaric acid ratio (*p*Cout/Caf) for each vineyard from treatment application to harvest in 2022 and 2023. Error bars represent standard errors (*n* = 3 and *n* = 4 for C1 in 2023). Different letters within the same column indicate significant differences between treatments. *p* ≤ 0.05; NS = not significant. The letters are presented vertically in order: C1, C0 (blue color), T1, T0 (orange color). S0–S5 represent sample points.

Skin HCTAs begin to accumulate early in berry development and are found in higher concentrations from bloom to veraison, at which point their concentration declines during the ripening period [67,68], as was observed in both years of this study (Figure 7).

In both years, HCTA total concentration was higher with UV deprivation, regardless of the thermal level, in parallel with the increase in *trans-p*-coutaric acid concentration, the predominant form of HCTA. Conversely, UV deprivation reduced the concentration of *cis-p*-coutaric acid (Supplementary Table S7). UV did not affect the concentration of *trans*-caftaric acid in 2022, but its concentration was reduced with increased UV exposure in 2023, resulting in a seasonal significant difference (Supplementary Table S7). The total HCTA concentration was not significantly influenced by the applied treatments at vineyard H in either year, whereas higher values in C0 than in T1 were observed at vineyards L and M in 2023. Grapes from vineyard L also accumulated higher amounts of HCTA in C0 with respect to C1 in 2022 (Figure 7).

Notably, all HCTA concentrations were significantly lower in 2023 as compared to 2022, reaching average values of 489.3 mg/kg of skin in 2022 and 448.6 mg/kg of skin in 2023 (Figure 7). As with flavonols, this aspect could be explained by the longer periods that grapes were exposed to extreme temperatures in 2023 with respect to 2022, assuming that the extreme peak of temperature could have blocked the first steps of the phenylpropanoid pathway when cinnamic acids are progressively synthesized [23]. Both the total HCTA and each individual HCTA showed significant differences between year and vineyard (Supplementary Table S7).

Ultraviolet radiation exposure influenced the ratio of *trans/cis p*-coutaric acid (Figure 7), with a decrease in *trans*-isomer with increased UV exposure and a corresponding increase in *cis p*-coutaric acid isomer. *Cis*-cinnamic acid is produced through a sunlight-mediated conversion from *trans*-cinnamic acid [69], and UV exposure serves to increase levels of *cis* isomers from *trans* isomers [70]. This response was consistent during the season and at harvest in all vineyards in both years (Figure 7). Globally, this aspect was more marked in 2023, which can suggest that the higher number of heat peaks and/or the higher SWC enhanced the conversion from *trans*-coutaric to *cis*-coutaric acid, particularly when cv Nebbiolo berries receive higher UV exposure. Considering that more than 50% of HCTA composition is comprised of *trans p*-coutaric acid, the ratio between *trans* and *cis* *p*-coutaric acid increased with the removal of UV in both years (Figure 7 and Supplementary Table S7). As the *cis* isomer of *p*-coutaric acid is less stable than the *trans* isomer, the alteration of this ratio could lead to a decreased concentration of *p*-coutaric and *p*-coumaric acid in wines. Further investigation into the effects of altering the ratio *trans/cis*-coutaric acid through increased UV exposure, thus reducing the total concentration of HCTA in berries, is required as a potential tool for risk reduction against possible spoilage from *Brettanoymyces* yeast [50].

Caftaric acid is very oxidizable and is predominantly accumulated in pulps where it reacts with glutathione, resulting in the GRP (grape reaction product, [71]). Considering that in red-cultivar winemaking, particularly in cv Nebbiolo grapes, the contribution of skin maceration to wine composition is important, and the contribution of HCTA concentration and profiles to wine quality cannot be neglected. For this reason, the ratio between *p*-coutaric acids (*cis* + *trans* forms) and caftaric acid (*trans*) was also calculated (Figure 7). Vineyards L and M displayed no significant differences in 2022, while in 2023, grapes from the L vineyard displayed higher values at S1, S2, and at harvest in treatment C0 with respect to T1 (Figure 7). Vineyard H did not show any difference during 2023, but differences were observed in 2022 from S4 to harvest with, again, C0 displaying a higher ratio than T1, suggesting that increased temperature can negatively influence this ratio, whereas lower temperature with reduced UV (C0) can increase the amount of *p*-coutaric acid compared to caftaric acid, leading to the lowered capacity of the wine to oxidize.

From this research, it appears that HCTA’s can be manipulated through alteration of UV exposure, while differences in thermal level did not show consistent responses. 

## 3. Materials and Methods

### 3.1. Experimental Site and Design

The research was performed in 2022 and 2023 on *Vitis vinifera* L. cv. Nebbiolo from veraison to harvest in three vineyards in the production region of Barolo wine (a wine that has a controlled and guaranteed designation of origin, DOCG) in Northwest Italy. It is a small area of approximately 80 km^2^, characterized by steep slopes and undulating hills ranging in elevation from just below 200 m above sea level (ASL) in the valley floor to 550 m ASL at a maximum elevation.

#### 3.1.1. Experimental Sites

**Figure 8:**
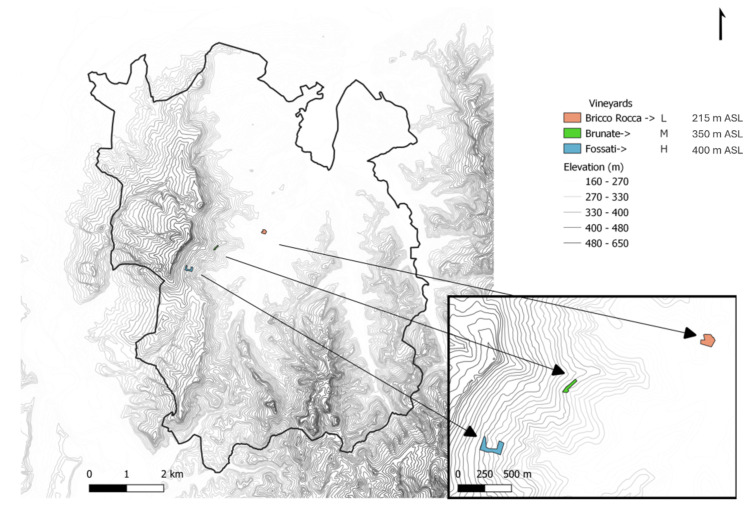
Map of Barolo DOCG production region (black border) with insert showing the location of the three vineyards in the study (Blue = H, Green = M, Pink = L). It should be noted that the 10 m resolution digital elevation model contours from the TINITALY digital elevation model [72].

The experiment was carried out in three non-irrigated vineyards at different elevations located in the municipality of La Morra (Piedmont Region, Italy). The lowest elevation vineyard (L) was located at 44°37′51.0″ N 7°57′21.5″ E in the Bricco Rocca site at an elevation of 215 m ASL; the middle elevation vineyard (M) was located at 44°37′39.4″ N 7°56′23.0″ E in Brunate site, at 350 m ASL; the highest elevation vineyard (H) was at 44°37′18.6″ N 7°55′48.6″ E in Fossati site at an elevation of 400 m ASL. The vineyards have ESE to SSE-facing slopes and similar slope gradients ranging from 12° to 15° (Figure 8). Vineyards M and H were planted in 2002 with S04 rootstock and clones CVT 141 and CVT 71, respectively. Vineyard L was planted in 1975 onto unknown rootstock with vines from massal selection. Vines were grown to a vertical shoot-positioned training system with single Guyot pruning (8 to 10 buds/vine). The rows were positioned along the contour lines. Vineyard soils were similar, with one major exception, as vineyard L had a much higher sand percentage than vineyards M or H (Supplementary Table S8).

#### 3.1.2. Experimental Design

**Figure 9:**
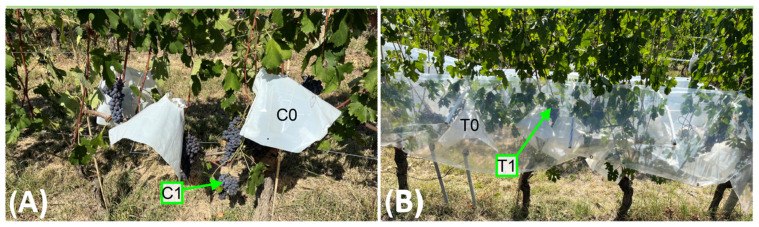
(**A**) Bunches exposed to ambient temperature with UV exposure (C1, no plastic cover) or exposed to ambient temperature and without UV exposure (C0, white UV-blocking plastic cover). (**B**) Bunches covered by passive plastic with UV exposure (T1) or without UV exposure (T0).

In each vineyard, three adjacent rows were chosen for a split-plot experimental design. The main factor, “temperature,” consisted of comparing the effects of two levels of temperature: the first level being ambient (C) and the second level being increased temperature (T), obtained by placing removable transparent plastic (Serroplast^®^, Rutigliano, BA, Italy) inducing a passive greenhouse effect. The greenhouse was designed to cover bunches of three consecutive vines per row. The greenhouse plastic was applied from the first training wire to cover the bunch zone but not to contact the ground. Curved rods were installed on the training wire perpendicular to the row orientation underneath the plastic to avoid direct contact between the leaves and the plastic (Figure 9B). The greenhouse plastic was connected above and below the bunch zone at several points to amplify temperature without completely closing the bunch zone.

The sub-plot factor “UV” consisted of testing two levels of UV radiation. To achieve this goal, a white UV-blocking plastic cover (Serroplast^®^) was applied (0) or not (1) over half of the grape bunches of each vine both inside the passive thermal treatment and outside (Figure 9A). Metal frames were shaped into a wide cone with a large end (bottom) approximately 40 cm in diameter and a small end (top) of 10 cm in diameter. These cones were covered in UV-blocking plastic with the top open. They were hung from the first training wire and suspended over individual bunches without contacting bunches or bunch rachis. The design allowed airflow in the bunched area to ensure temperature was minimally influenced. Measurements of transmitted UVA, UVB, and PAR were acquired for each treatment under midday full sun conditions (5 min per treatment at 10 sec/sample) to determine differences between treatments (Delta Ohm DO9847, GHM Group, Regenstauf, Germany).

Four treatments were then compared: ambient temperature and full UV exposure (C1), ambient temperature and no UV exposure (C0), amplified temperature and full UV exposure (T1), and amplified temperature and no UV exposure (T0) (Figure 9). Each vineyard had three replicates per treatment (four replicates for C1 treatment in 2023) and three vines per replicate, for a total of 18 vines per vineyard. The four treatments were applied in all vineyards when berries began to develop color at BBCH 81 [73]: 25 July 2022, and 4 August 2023. The experimental design was randomized with limitations based on weak production levels (this was particularly true for the “T” treatments, which required multiple adjacent vines with a minimum of 4 bunches per vine for the greenhouse treatment to cover a suitable number of replicates for both T1 and T0), and disease presence in both years.

### 3.2. Air and Soil Temperature, Soil Volumetric Water Content, and Precipitation Assessment

Air temperature was acquired in C and T treatments during the research period in both seasons. In July 2022, one temperature sensor (HOBO Datalogger MX2301A—Onset Computer Corporation, Bourne, MA, USA) was installed in the bunch zone in the center of the middle row in each vineyard. This temperature sensor remained on site, reading ambient temperature (C treatment) until the end of harvest 2023. At the time of treatment application, a second temperature sensor was installed in the middle row under the passive greenhouse (T treatment) in each vineyard at the same height as the C temperature sensor (HOBO Datalogger MX2302—Onset Computer Corporation) and Tinytag Plus 2 TGP 4500 (Gemini Data Loggers Ltd., Chichester, UK), which measured the hourly minimum daily temperature (TMin) and maximum daily temperature (TMax). A partial Huglin Index (HI) was calculated for each treatment from August 23 to October 8 in both years. The standard HI (from 1 April to 31 October) could not be calculated because sensors were installed in the vineyards in late July 2022, and some data were lost due to anomalies in data recording, particularly during the months of August and September 2023.

An assessment was also carried out inside a UV treatment cone (C0) and outside (C1) from mid-May to mid-July of 2022 to determine whether the air temperature could be amplified under the UV-blocking plastic.

Soil volumetric water content (SWC) and soil temperature were measured in each vineyard with a “5TM Soil Moisture and Temperature Sensor” equipped with an EM 50 Datalogger (Decagon Devices, Inc., Pullman, WA, USA). SWC sensor probes were installed in the middle row of the experimental row group into the undisturbed sidewall of the borehole at 30 cm depth on 6 June 2022 and operated until 15 October 2023. A second SWC sensor was installed in each vineyard, 1 m from the first sensor, prior to the commencement of 2023 activities as backup sensors. Soil sensors were only installed in C treatment. The temperature and precipitation at the meso-scale were obtained from a nearby weather station (La Morra, LM, Italy, at 326 m ASL) [74].

### 3.3. Berry Sampling and Berry Skin Preparation

In both years, berries from each treatment were sampled randomly from both sides of all rows, with 10 berries per replicate, 3 replicates per treatment in each vineyard, and 4 replicates collected for C1 treatment in 2023. In 2022, treatments were applied on 25 July at the first sign of veraison. Berry sampling commenced at an estimated 50% veraison (4 August (S1)). A second sample was collected one week later at 100% veraison (12 August (S2)); 2 intermediate samples were taken (29 August (S3) and 11 September (S4)) prior to the final sample at harvest (26 September (M); 1 October (H); 3 October (L) (S5)). In 2023, C1 samples were collected on the day of treatment application (4 August (S0)). Samples were then collected at approximately 2-week intervals until harvest (18 August (S1), 1 September (S2), 15 September (S3), and 29 September (S4)), and at harvest (9 October (M); 12 October (H); 15 October (L) (S5)). The final harvest samples were collected the day prior to commercial harvest in each vineyard in both years.

Berries were cut above the pedicel, placed in a sealed plastic bag, and stored in a portable refrigerator until they could be transported to the laboratory (within 1 h). At the laboratory, fresh berries were weighed (BFW), and then pedicels were removed, with pulp separated from the berry skin. A tight sealing container with 40 mL of 3.2 pH buffer solution (120 mL/L ethanol, 5 g/L tartaric acid, 2 g/L Na_2_S_2_O_5,_ 22 mL/L NaOH, 1 mol/L) was weighed, skins were immediately added, and then the container was weighed again to determine skin fresh weights (SFW); the ratio skin weight–berry weight was calculated. Berry skins in buffer were frozen at −20 °C. Pulps were preserved to measure total soluble solids (TSS, Brix) by a refractometer (HI96811, Hanna Instruments, Woonsocket, RI, USA), which was then converted to grams per berry. Berry skin extracts were thawed and homogenized twice (UltraTurrax T25, IKA, Staufen, Germany) and centrifuged for 15 min at 2220× *g* (Heraeus Primo, Thermo Fisher Scientific, Boston, MA, USA), taking the extracts to a known final volume (50 mL). Extracts were stored in tightly sealed 50 mL plastic containers and frozen prior to preparation for high-performance liquid chromatography (HPLC-DAD) analysis.

### 3.4. Anthocyanin, Flavonol, and Hydroxycinnamic Tartaric Acids Extract Preparation and Chromatographic Analyses

Samples were prepared for anthocyanin and flavonol/HCTA analyses according to a method modified by Di Stefano and Cravero (1991) [75].

Anthocyanins were detected by HPLC/DAD analysis using Agilent 1200 series equipment (Agilent Technologies, Santa Clara, CA, USA) equipped with a LiChrospher^®^ 100 RP-18 (5 µm particle size, 25 cm × 0.4 cm ID) (Merck, Darmstadt, Germany) column. Formic acid–water (10:90, *v*/*v*) and formic acid–methanol–water (10:50:40, *v*/*v*/*v*) were used as solvents A and B, respectively. A linear gradient between 28% and 45% of solvent B over 15 min, then to 70% in 20 min, and finally to 90% in 10 min was used for the separation. The column was then washed with solvent B for 3 min before returning to the starting condition (28% B) for 10 min. A constant flow rate of 0.8 mL/min was established. Detection was carried out at 520 nm wavelength [75].

Delphinidin (Df), Cyanidin (Cy), Petunidin (Pt), Peonidin (Pn), and Malvidin (Mv) 3-*O*-glucosides (Gluc) were detected as well as their relative acylated forms: acetated anthocyanins (Acet) and *p*-Coumaroylated anthocyanins (*p*Coum). The ratios Pn/Cy and Mv/(Dp + Pt) were calculated to estimate the degree of methoxylation of di- and tri-hydroxylated anthocyanin, respectively. The identification and quantification of the individual anthocyanins was based on the comparison of their retention time with that of pure standards, when available, and the concentration was expressed as malvidin 3-*O*-glucoside equivalents (Extrasynthèse, Genay, France).

Samples for flavonol and HCTA analysis were processed after dilution with 1 mol/L phosphoric acid. HPLC analysis was performed using an Agilent 1260 Infinity System (Agilent Technologies). Solvent A (phosphoric acid 10^−3^ mol/L) and solvent B (CH_3_OH) were used, applying gradient elution conditions starting with 5% B, increasing linearly to 100% B in 35 min, and keeping 100% B for 5 min, followed by a re-equilibration phase under isocratic conditions. The flow rate was 0.8 mL/min, and chromatographic acquisitions were set at 360 and 320 nm. Flavonol concentrations were expressed as quercetin dehydrated equivalent per kilogram of fresh berries. Among flavonols, myricetin 3-*O*-glucoside (MSide), quercetin 3-*O*-glucoside (QSide), quercetin 3-*O*-glucuronide (QRide), kaempferol 3-*O*-glucoside (KSide), and kaempferol 3-*O*-glucuronide (KRide) were identified based on previous published papers [14,76] and quantified as quercetin 3-*O*-glucoside equivalents (Extrasynthèse). The ratio of glucoside forms (Side) to glucuronides (Ride) was calculated to evaluate the relative abundance of the prevalent class of flavonol-glycosides.

HCTA chromatograms were acquired at 320 nm and expressed as equivalents of caftaric acid per kilogram of skins. Among HCTAs, *trans* caftaric acid and *cis* and *trans p*-coutaric acid were identified.

The total concentration of each class of compounds was obtained by summing the individual concentrations.

### 3.5. Statistical Analysis

A generalized linear model (GLM) was used to investigate the effects of the treatments by year and vineyard, including their interactions. Prior to running the GLM, normality (Shapiro–Wilk test) and homoscedasticity (Breusch–Pagan test) were assessed. Post hoc analysis was performed using estimated marginal means (EMMs) to explore pairwise comparisons among the levels of the factors and vineyards. The contrasts were adjusted for multiple comparisons using false discovery rate correction. Statistical significance was assessed at *p* ≤ 0.05.

A one-way ANOVA was performed on temperature data between C1 and C0 treatments to determine if there was a significant temperature amplification in C0 (and, by extension, T0) as compared to C1 and T1.

Statistical analysis was performed with the statistical software R [77] with multcomp [78] and emmeans packages [79] using RStudio GUI [80]. Graphical representation of plots was produced with Microsoft Excel version 2410 (Microsoft Corporation, Redmond, WA, USA).

## 4. Conclusions

At the field level, interactions between cultivar and environment are complex. Both temperature and UV exposure can impact the development of berry characteristics and polyphenols and, thus, the end quality and identity of a wine. In a changing climate, producers will have to consider curating management based on vineyard location, cultivar, clonal characteristics, current local risks to berry and wine quality, and desired qualitative features of the wine. In the case of cv Nebbiolo, UV plays a significant role in color development and stability due to its high concentration of di-hydroxylated anthocyanins, which have been shown to be both temperature- and UV-sensitive. Increasing the percentage incidence of the tri-hydroxylated malvidin 3-*O*-glucoside through reduced UV exposure could increase wine color stability; the reduction in UV exposure also leads to decreased flavonol and increased HCTA concentrations. Increased temperature has long been associated with decreased anthocyanin concentration, and in this research, similar observations were made. The combination of increased temperature and decreased UV exposure further amplified this decline in cv Nebbiolo grapes, suggesting that cultivars with a specific anthocyanin profile characterized by higher concentrations of di-hydroxylated anthocyanins can be manipulated through UV exposure as well as temperature. The effect of temperature on flavonol concentrations has long been debated, but in this research, given the passive nature of the treatments and the significant difference between the number of hours of exposure to extreme temperatures (>40 °C), flavonols were notably lower in treatments with increased temperature. Although this research considered only the effects of UV exposure and temperature on some berry characteristics and flavonoids in berry skin, other factors also play a role in berry quality. Consideration of the hill aspect, as well as soil water retention, along with other site-specific details, must also be factored into the decision-making process for an appropriate vineyard management strategy to balance UV exposure and temperature for desired outcomes in berry and wine quality.

## Figures and Tables

**Table 1:** Ultraviolet A (UVA), ultraviolet B (UVB), and photosynthetically active radiation (PAR) spectral ranges penetrating vine canopy of treated vines compared to control (C1).

Treatment	UVA (%)	UVB (%)	PAR (%)
C1	100	100	100
C0	7	1	53
T1	76	68	86
T0	5	1	49

**Table 2:** Huglin Index (HI) from August 23 to October 8 in 2022 and 2023 at the vineyard level. Number of hours (Hrs) above 35 °C, 40 °C, and 45 °C from Aug 4 to harvest in both years. C = control treatment, T = temperature increased treatment.

	Vineyard	2022		2023	
		C	T	C	T
HI	L	745.8	896.8	843.5	961.0
	M	827.1	914.9	796.9	960.8
	H	763.6	925.3	854.8	1000.0
Hrs > 35 °C	L	34	211	157	248
	M	119	191	92	202
	H	61	172	163	200
Hrs > 40 °C	L	0	61	40	127
	M	8	19	27	46
	H	0	61	42	66
Hrs > 45 °C	L	0	11	0	37
	M	0	0	0	4
	H	0	2	0	12

## Data Availability

The raw data supporting the conclusions of this article will be made available by the authors upon request.

## Supplementary Materials

The following supporting information can be downloaded at: https://www.mdpi.com/article/10.3390/plants13223158/s1, Table S1: Berry characteristics at harvest: Berry Fresh Weight (BFW), Skin Fresh Weight (SFW), Skin-to-Berry Ratio, Total Soluble Solids (TSS), and Sugar per Berry at harvest (S5); Table S2: Anthocyanin concentration at harvest for the glucosylated forms: Delphinidin (Df), Cyanidin (Cy), Petunidin (Pt), Peonidin (Pn) and Malvidin (Mv) at harvest (S5); Table S3: Anthocyanin proportion at harvest for glucosylated forms: Delphinidin (Df), Cyanidin (Cy), Petunidin (Pt), Peonidin (Pn) and Malvidin (Mv) at harvest (S5); Table S4: Total Anthocyanin Concentration (TAC); Di-hydroxylated/Tri-hydroxylated ratio (Di/Tri); Acylated/Total Anthocyanins ratio (Acyl/TAC); Rate of methoxylation in di-hydroxylated anthocyanins {Di-Meth = Pn/Cy}); Rate of methoxylation in tri-hydroxylated anthocyanins, [Tri-Meth = Mv/(Df + Pt)]; Ratio between TAC and Total Soluble Solids (TAC/TSS) at harvest (S5); Table S5: Individual flavonol proportion (% of total) at harvest. Myricetin 3-*O*-glucoside (MSide); Quercetin-3-*O*-glucuronide (QRide); Quercetin-3-*O*-glucoside (QSide); Kaempferol-3-*O*-glucuronide (KRide); Kaempferol-3-*O*-glucoside (KSide) at harvest (S5); Table S6: Total and individual flavonols concentration (mg/kg) and Glucoside/Glucuronide ratio at harvest. Myricetin 3-*O*-glucoside (MSide); Quercetin-3-*O*-glucuronide (QRide); Quercetin-3-*O*-glucoside (QSide); Kaempferol-3-*O*-glucuronide (KRide); Kaempferol-3-*O*-glucoside (KSide); Total Glucoside/Total Glucuronide ratio (Side/Ride) at harvest (S5); Table S7: Concentration of total and individual Hydroxycinnamic acid (HCTA), *trans* Caftaric acid (*trans* Caf), *cis p*Coutaric acid (*cis p*Cou), *trans p*Coutaric acid (*trans p*Cou), Total *p*Coutaric forms (*cis* + *trans p*Cou), ratio *trans/cis* forms of *p*Coutaric acid (*trans/cis p*Cout) and ratio *p*Coutaric acid/Caftaric acid (*p*Cout/Caf) at harvest (S5); Table S8: Soil characteristics from a soil sample either proximate to the vineyard of research (L) or in the vineyard of research (M and H).
