# Supplementary material for: The Effect of Temperature and UV Manipulation on Anthocyanins, Flavonols, and Hydroxycinnamoyl-Tartrates in cv Nebbiolo Grapes (Vitis vinifera L.)
Source: Plants (Basel). 2024 Nov 10;13(22):3158. doi: 10.3390/plants13223158 (PMC11597326; doi:10.3390/plants13223158)
Supplement: Supplementary file 1 [file plants-13-03158-s001.zip › plants-3278746-supplementary.pdf]

plants

MDPI

Supplementary Data

Supplementary Table S1: Berry characteristics at harvest: Berry Fresh Weight (BFW), Skin Fresh Weight (SFW), Skin to Berry Ratio, Total Soluble Solids (TSS) and Sugar per Berry at harvest (S5). The significance between treatments, vineyards and years and of the interactions between factors is also reported.

Treatment	BFW (g)	SFW (g)	Skin to Berry Ratio	TSS (°Brix)	Sugar per Berry (g/berry)
C1[§]	1.499 a[1]	0.118 a	0.079 b	24.9 ab	0.37 a
C0	1.403 b	0.111 a	0.080 b	25.2 a	0.35 ab
T1	1.402 b	0.119 a	0.084 ab	24.7 b	0.34 b
T0	1.283 c	0.111 a	0.087 a	24.6 b	0.31 c
Vineyard					
L[†]	1.694 a	0.134 a	0.079 b	24.3 b	0.41 a
M	1.144 c	0.102 b	0.089 a	25.6 a	0.29 c
H	1.353 b	0.108 b	0.081 b	24.6 b	0.33 b
Significance					
Year	NS[2]	***	***	***	NS
Vineyard	***	***	***	***	***
Treatment	***	*	*	**	***
Year*Vineyard	***	***	***	*	***
Year*Treatment	NS	NS	NS	**	**
Vineyard*Treatment	NS	NS	NS	NS	NS
Year*Vineyard*Treatment	***	NS	NS	*	***

[§]The values are the average between years and vineyards or [†]between treatments and years.
[1]For each treatment and vineyard, different letters indicate significant differences with $p \leq 0.05$. [2] *** ($p < 0.0001$); ** ($p < 0.001$); * ($p < 0.05$); NS = Not Significant. Results were determined from general linear model (GLM) with post-hoc analysis based on estimated marginal means (EMMs), with false discovery rate correction.

Supplementary Table S2: Anthocyanin concentration at harvest for the glucosylated forms: Delphinidin (Df), Cyanidin (Cy), Petunidin (Pt), Peonidin (Pn) and Malvidin (Mv) at harvest (S5). The significance between treatments, vineyards and years and of the interactions between factors is also reported.

Treatment	Df (mg/kg)	Cy (mg/kg)	Pt (mg/kg)	Pn (mg/kg)	Mv (mg/kg)
C1[§]	20.7 a[1]	99.0 a	28.4 a	251.5 a	112.9 a
C0	19.0 a	71.0 b	28.3 ab	212.5 b	118.9 a
T1	17.8 a	74.1 b	24.8 b	185.1 c	97.0 b
T0	18.5 a	55.0 c	25.1 ab	166.7 c	106.0 ab
Vineyard					
L[†]	21.2 a	66.0 b	24.1 b	188.2 b	92.2 b
M	24.0 a	91.3 a	30.1 a	230.7 a	114.2 a
H	11.8 b	67.0 b	25.8 b	192.9 b	119.7 a
Significance					
Year	NS[2]	***	NS	NS	***
Vineyard	***	***	***	***	***
Treatment	NS	***	**	***	***
Year*Vineyard	*	***	**	***	NS
Year*Treatment	NS	NS	*	NS	NS
Vineyard*Treatment	NS	NS	NS	NS	NS
Year*Vineyard*Treatment	NS	**	NS	*	NS

[§]The values are the average between years and vineyards or [†]between treatments and years.
[1]For each treatment and vineyard, different letters indicate significant differences with $p \leq 0.05$; [2]*** ($p < 0.0001$); ** ($p < 0.001$); * ($p < 0.05$); NS = Not Significant. Results were determined from general linear model (GLM) with post-hoc analysis based on estimated marginal means (EMMs), with false discovery rate correction.

Supplementary Table S3: Anthocyanin proportion at harvest for glucosylated forms: Delphinidin (Df), Cyanidin (Cy), Petunidin (Pt), Peonidin (Pn) and Malvidin (Mv) at harvest (S5). The significance between treatments, vineyards and years and of the interactions between factors is also reported.

Treatment	Df (%)	Cy (%)	Pt (%)	Pn (%)	Mv (%)
C1§	3.3 a[1]	15.7 a	4.6 b	40.4 a	18.5 b
C0	3.3 a	12.4 c	5.0 a	37.7 b	21.6 a
T1	3.4 a	14.3 b	4.9 ab	36.0 c	19.3 b
T0	3.7 a	11.3 c	5.2 a	34.7 c	22.3 a
Vineyard					
L†	4.2 a	13.2 b	4.9 a	37.4 a	18.7 b
M	3.9 a	14.4 a	4.9 a	36.9 a	18.8 b
H	2.3 b	12.8 b	5.1 a	37.2 a	23.7 a
Significance					
Year	**[2]	***	***	NS	***
Vineyard	***	**	NS	NS	***
Treatment	NS	***	**	***	***
Year*Vineyard	NS	***	***	***	***
Year*Treatment	NS	NS	*	NS	NS
Vineyard*Treatment	NS	*	NS	NS	NS
Year*Vineyard*Treatment	NS	NS	*	NS	NS

§The values are the average between years and vineyards or †between treatments and years.

[1]For each treatment and vineyard, different letters indicate significant differences with $p \leq 0.05$; [2]*** ($p < 0.0001$); ** ($p < 0.001$); * ($p < 0.05$); NS = Not Significant. Results were determined from general linear model (GLM) with post-hoc analysis based on estimated marginal means (EMMs), with false discovery rate correction.

Supplementary Table S4: Total Anthocyanin Concentration (TAC); Di-hydroxylated/Tri-hydroxylated ratio (Di/Tri); Acylated/Total Anthocyanins ratio (Acyl/TAC); Rate of methoxylation in di-hydroxylated anthocyanins {Di-Meth = Pn/Cy}); Rate of methoxylation in tri-hydroxylated anthocyanins, [Tri-Meth = Mv/(Df+Pt)]; Ratio between TAC and Total Soluble Solids (TAC/TSS) at harvest (S5). The significance between treatments, vineyards and years and of the interactions between factors is also reported.

Treatment	TAC (mg/kg)	Di/Tri	Acyl/TAC	Di-Meth (mg/kg	Tri-Meth (mg/kg)	TAC/TSS
C1§	621.3 a[1]	2.22 a	0.18 c	2.65 b	0.45 a	24.9 a
C0	562.7 b	1.74 bc	0.20 b	3.14 a	0.41 a	22.3 b
T1	511.7 bc	1.86 b	0.22 a	2.57 b	0.44 a	20.7 bc
T0	480.6 c	1.51 c	0.23 a	3.19 a	0.42 a	19.5 c
Vineyard						
L†	498.1 b	1.85 a	0.22 a	2.90 a	0.49 a	20.5 b
M	620.6 a	1.99 a	0.21 a	2.67 b	0.48 a	24.2 a
H	513.5 b	1.66 b	0.19 b	3.10 a	0.32 b	20.8 b
Significance						
Year	NS[2]	***	***	***	**	NS
Vineyard	***	***	***	***	***	***
Treatment	***	***	***	***	*	***
Year*Vineyard	***	***	NS	**	***	***
Year*Treatment	NS	NS	NS	NS	NS	NS
Vineyard*Treatment	NS	NS	NS	*	NS	NS
Year*Vineyard*Treatment	NS	**	NS	NS	NS	NS

§The values are the average between years and vineyards or †between treatments and years.
[1]For each treatment and vineyard, different letters indicate significant differences with $p \leq 0.05$; [2]*** ($p < 0.0001$); ** ($p < 0.001$); * ($p < 0.05$); NS = Not Significant. Results were determined from general linear model (GLM) with post-hoc analysis based on estimated marginal means (EMMs), with false discovery rate correction.

Supplementary Table S5: Individual flavonol proportion (% of total) at harvest. Myricetin 3-O-glucoside (MSide); Quercetin-3-*O*-glucuronide (QRide); Quercetin-3-*O*-glucoside (QSide); Kaempferol-3-*O*-glucuronide (KRide); Kaempferol-3-*O*-glucoside (KSide) at harvest (S5). The significance between treatments, vineyards and years and of the interactions between factors is also reported.

Treatment	MSide (%)	QRide (%)	QSide (%)	KRide (%)	KSide (%)
C1§	3.7 c[1]	15.3 c	65.1 a	2.7 a	12.3 a
C0	7.0 a	32.7 a	54.2 c	0.8 c	4.2 c
T1	4.8 b	18.8 b	63.4 b	2.1 b	10.3 b
T0	7.5 a	32.5 a	53.7 c	0.9 c	4.1 c
Vineyard					
L†	6.0 a	23.6 b	59.5 a	2.1 a	7.9 a
M	5.5 a	24.2 b	60.4 a	1.3 b	7.7 a
H	5.9 a	26.6 a	57.3 b	1.5 b	7.6 a
Significance					
Year	NS[2]	***	*	***	***
Vineyard	NS	***	***	***	NS
Treatment	***	***	***	***	***
Year*Vineyard	***	***	***	***	**
Year*Treatment	NS	NS	*	NS	*
Vineyard*Treatment	**	NS	*	***	NS
Year*Vineyard*Treatment	NS	**	NS	***	*

§The values are the average between years and vineyards or †between treatments and years.
[1]For each treatments and vineyards, different letters indicate significant differences with $p \leq 0.05$; [2]*** ($p < 0.0001$); ** ($p < 0.001$); * ($p < 0.05$); NS = Not Significant. Results were determined from general linear model (GLM) with post-hoc analysis based on estimated marginal means (EMMs), with false discovery rate correction.

Supplementary Table S6: Total and individual flavonols concentration (mg/kg) and Glucoside/Glucuronide ratio at harvest. Myricetin 3-O-glucoside (MSide); Quercetin-3-*O*-glucuronide (QRide); Quercetin-3-*O*-glucoside (QSide); Kaempferol-3-*O*-glucuronide (KRide); Kaempferol-3-*O*-glucoside (KSide); Total Glucoside/Total Glucuronide ratio (Side/Ride) at harvest (S5). The significance between treatments, vineyards and years and of the interactions between factors is also reported.

Treatment	Total Flavonols (mg/kg)	MSide (mg/kg)	QRide (mg/kg)	QSide (mg/kg)	KRide (mg/kg)	KSide (mg/kg)	Side/Ride
C1[§]	150.1 a[1]	5.5 a	23.0 a	97.6 a	4.1 a	18.5 a	4.6 a
C0	58.9 c	4.0 b	19.0 b	32.2 c	0.5 c	2.6 c	2.0 c
T1	122.4 b	5.8 a	22.9 a	77.7 b	2.6 b	12.7 b	3.8 b
T0	56.3 c	4.2 b	18.0 b	30.4 c	0.5 c	2.4 c	2.0 c
Vineyard							
L[†]	92.2 a	4.7 a	18.6 b	57.2 a	2.1 a	8.8 a	3.3 a
M	102.5 a	5.1 a	22.1 a	63.6 a	1.7 a	9.2 a	3.1 a
H	96.1 a	4.9 a	21.6 a	57.6 a	1.9 a	9.1 a	2.9 b
Significance							
Year	NS[2]	NS	NS	NS	**	***	***
Vineyard	NS	*	***	NS	NS	NS	***
Treatment	***	***	***	***	***	***	***
Year*Vineyard	NS	**	NS	**	**	NS	***
Year*Treatment	*	**	**	*	NS	*	*
Vineyard*Treatment	NS	**	*	NS	*	NS	*
Year*Vineyard*Treatment	NS	*	NS	NS	NS	NS	NS

[§]The values are the average between years and vineyards or [†]between treatments and years.
[1]For each treatment and vineyard, different letters indicate significant differences with $p \leq 0.05$; [2]*** ($p < 0.0001$); ** ($p < 0.001$); * ($p < 0.05$); NS = Not Significant. Results were determined from general linear model (GLM) with post-hoc analysis based on estimated marginal means (EMMs), with false discovery rate correction.

Supplementary Table S7: Concentration of total and individual Hydroxycinnamic acid (HCTA), *trans* Caftaric acid (*trans* Caf), *cis p*Coutaric acid (*cis p*Cou), *trans p*Coutaric acid (*trans p*Cou), Total *p*Coutaric forms (*cis* + *trans p*Cou), ratio *trans/cis* forms of *p*Coutaric acid (*trans/cis p*Cout) and ratio *p*Coutaric acid/Caftaric acid (*p*Cout/Caf) at harvest (S5). The significance between treatments, vineyards and years and of the interactions between factors is also reported.

Treatment	HCTA (mg/kg)	*Trans* Caf (mg/kg)	*cis p*Cou (mg/kg)	*trans p*Cou (mg/kg)	*cis* + *trans p*Cou (mg/kg)	*Trans/Cis p*Cout	*p*Cout/Caf
C1[§]	456.3 bc[1]	120.3 a	55.2 a	256.3 b	311.6 bc	4.6 c	2.7 b
C0	508.5 a	126.7 a	48.2 b	309.5 a	357.7 a	6.4 a	2.9 a
T1	425.7 c	113.2 a	54.3 a	236.9 b	291.2 c	4.4 c	2.7 b
T0	485.3 ab	124.7 a	49.1 b	290.0 a	339.0 ab	5.9 b	2.8 ab
Vineyard							
L[†]	419.0 b	97.8 c	47.8 b	250.8 b	298.6 b	5.3 b	3.1 a
M	488.5 a	140.3 a	51.0 b	282.8 a	333.8 a	5.6 a	2.5 c
H	499.3 a	125.5 b	56.3 a	286.0 a	342.3 a	5.1 b	2.7 b
Significance							
Year	**[2]	***	*	NS	NS	*	***
Vineyard	***	***	***	***	***	***	***
Treatment	***	NS	***	***	***	***	***
Year*Vineyard	***	***	***	***	***	**	***
Year*Treatment	NS	NS	NS	NS	NS	NS	NS
Vineyard*Treatment	NS	NS	NS	NS	NS	**	NS
Year*Vineyard*Treatment	NS	NS	NS	NS	NS	NS	*

[§]The values are the average between years and vineyards or [†]between treatments and years.
[1]For each treatment and vineyard, different letters indicate significant differences with $p \leq 0.05$; [2]*** ($p < 0.0001$); ** ($p < 0.001$); * ($p < 0.05$); NS = Not Significant. Results were determined from general linear model (GLM) with post-hoc analysis based on estimated marginal means (EMMs), with false discovery rate correction.

Supplementary Table S8: Soil characteristics from a soil sample either proximate to the vineyard of research (L) or in the vineyard of research (M and H).

	H	M	L
Sand %	9.2	8.6	48.0
Silt %	57.9	62.3	30.0
Clay %	32.9	29.1	22.0
pH	8.2	8.3	8.2
Total Carbonate	22.6	23.3	23.0
Total Nitrogen	0.098	0.075	0.049
CEC	15.7	12.0	nd
